# Country Holidays for Town Children

**Published:** 1896-09-19

**Authors:** 


### Modern Sociology.

### COUNTRY HOLIDAYS FOR TOWN CHILDREN.

One most useful form of charity claims special notice —a form which, like most others, has its abuses as well as its uses. It was a happy thought which first suggested that a week or two spent in the fresh country air, amid rural scenes so delightful yet so strange to the town child, could not fail to have a bracing effect on body and mind of the pallid little ones whose life is spent in close courts and narrow streets. Sick and convalescent children had many openings by which they could reach the benefit and delights of country air and scenes, which were still denied to others. Now, thanks to the numerous holiday fund societies, many thousands of London children enjoy yearly a fortnight in breezy country air, and taste the, to them, rapturous delights of "fresh fields and pastures new." Some get but one day—a day to be marked with a white stone, when they sing—

> "In the sweet lanes, on country wains,
> 'Mid honeysuckle, heather,
> Roses and hay, fragrant and gay,
> We bask in summer weather."

But the adoption of the plan of boarding the children with country folk in cottages enables the societies to give the advantage of a real holiday to thousands at a very moderate cost.

The objects and methods of these societies were thus summed up, with admirable lucidity and brevity, by the Bishop of London in a letter to the *Times* :—

> "Many of your readers already recognise the advantage of fresh air for London children, and subscribe the 10s. which secures for one out of the 750,000 in the elementary schools a fortnight in a cottager's home. The children are selected from each school, voluntary or Board, by visitors, who establish personal relations with the children; they are sent to cottagers as guests in the family, and thus get a variety of interests and form friendships with the country people hardly possible in any sort of institution. The cottages are selected and supervised by responsible residents in the country. The money subscribed is apportioned to each London district according to its needs in such a way that the poorer parts obtain their full share of the fund. The parents are asked to make the holiday their own affair by bearing whatever proportion of the cost they can afford. Last year money was liberally given, and 28,783 children were sent. Large as this number seems, it is but small when compared with those left behind. I hope that this year the need of these London little ones will not be forgotten, and that those who are about to take holiday themselves will send a donation to the Hon. Alfred Lyttelton, M.P., the treasurer, at 10, Buckingham Street, Strand, W.C."

Doubtless an appeal from such a source for so admirable an object, carried out by a society as well-known as efficient, will always meet with a hearty response, for subscribers to Mr. Lyttelton's fund may feel perfect confidence that each 10s. will actually give a fortnight in the country to some child who needs the change.

But, unhappily, there are rogues who intercept and turn into their own reservoirs even this innocent rill of the blessed stream of charity, either by a direct and barefaced appropriation of the whole, or by ostentatiously allowing a small part to be used for its proper purpose, whilst they surreptitiously abstract the rest.

An instance of the barefaced robbery of the whole may be found in a case tried before Mr. Sheil at the Westminster Police Court on June 26th this year. A Mrs. Heritage, who kept a lodging-house and two servants, was convicted of obtaining charitable contributions by fraud. She had represented that she was an authorized collector of funds for summer treats for school children. Armed with a "Court Guide" and books containing lists of subscriptions, she went from house to house in Belgravia, and, as Mr. Sheil remarked, "had, apparently from her books, cheated half the peerage." Her operations extended over two years, during which she had collected (and spent) over £200.

Mr. Sheil sentenced this goodly Heritage to nine months' imprisonment, with hard labour, remarking, "It is a most wicked, cruel, and abominable fraud, enough to dry up the stream of charity all over the country. It is a great deal worse than stealing the money."

Nor is this a solitary case. Several of a like character, in which the appeals were made on behalf of Children's Fresh-Air Mission and kindred societies, have been brought into the police-courts this summer. The plea is chosen by impostors because of its peculiar force. Few would refuse to give of their abundance a trifle to send the poor children of the great city into the fields which many of them have never seen. The amount at present contributed is respectable, but it might easily be increased tenfold if donors would take the trouble to send their gifts direct to the societies. This is the only way to avoid being imposed upon, and to make sure that the poor children benefit by the gift.

In this, as in every other class of charities, there are societies and societies. Some few "one-man" arrangements are, we fear, as barefaced robbers as the Heritage, though they manage to avoid legal consequences by astute quibbles. Others do not deserve support because they spend more on official expenses than on the children. Thus, whereas the Children's Country Holiday Fund is managed at a cost of barely 6 per cent. of the subscriptions, the expenses of administration of Mr. Briton's Southend Holiday Home amount, as we showed a few weeks ago, to 58 per cent.

Mr. C. S. Loch has done good service by his timely letters to the *Times* last week, proposing a conference of all who are genuinely working to secure reasonable country holidays for poor City children. We hope the conference will be speedily held, and end in the removal of the existing abuses. In any case, every generous donor will be well advised to adopt the following suggestions :—

1. By all means give. It is a worthy cause which should be more widely supported.

2. Send your contribution direct to the society, but, before doing so, unless it is a well-known society such as the Children's Country Holiday Fund, 10, Buckingham-street, W.C., make inquiries and ask for a copy of the last report.

3. In case of doubt, write for information to the Charity Organisation Society, 15, Buckingham Street, W.C.

